# Extraction of a Tooth

**Published:** 1870-07

**Authors:** S. P. Cutler


### EXTRACTION OF A TOOTH.

BY S. P. CUTLER, M. D. D. D. S.

Professor of Chemistry in the New Orleans Dental College.

Some fifteen years ago a country physician called at my office with a young man, about seventeen years old, for the purpose of having a tooth examined.

The young man, a farm laborer, was pale, emaciated, sallow, and bloated in the face; had been suffering from chills and fever for a number of weeks, with neuralgia of one side of his face, which had resisted all remedial efforts. He was very much reduced in flesh and strength, and his appetite was capricious.

On examining his mouth I found the superior anterior molar of right side much decayed, and on making the usual tests found the tooth dead, and sore to the touch, increasing pain in cheek when taped with an instrument.

I at once decided on extraction, which I did with some difficulty. The tooth had longer and more forked roots than any tooth I ever saw before or since.

To one of the buccal roots there was attached a sack about one inch in length, and about one-half inch in diameter, partly filled with matter, about the consistency of pus. The attachments of the sack embraced the entire end of root.

My curiosity being excited I introduced a probe into the corresponding socket, which readily passed into the antrum, and explained the whole mystery of the case.

In the course of three or four weeks, without any further treatment, he entirely recovered his health. All local and general symtoms disappearing.

Sacks never form at the ends of roots of live teeth, or until after the death of pulp. In the above case the root projected far into the antrum, and there the sack was formed around the point of the root, completely enveloping the point, forming a closed sack, and, as a matter of necessity, in communication with the pulp cavity. How could the matter in the sack get there, unless the sack itself secreted from the epithelia of the inner walls the decomposed pulp, no doubt, forming a portion? How are such sacks nourished? Is it by osmosis, or by vessels? We certainly have no definite data on this subject. These sacks grow from exterior nutrition, though an endogenous action, most likely. We are not quite sure of this fact, however, especially in the above named case, as it was situated in the antrum which is itself lined with a periosteal membrane quite different from the alveolar periosteum where similar sacks are frequent. Now then in a physiological state these periosteums perform both similar and dissimilar functions; in the one case furnishing nutrition to the alveolus and cement; in the other furnishing nutrition to the bone, and a lubricating fluid from true epithelia, similar to the mucous surfaces of the mouth.

In a morbid, or pathological condition these bone membranes furnish identically the same kind of tumors, and no doubt are furnished in the same way, whether it be by osmosis or direct from blood vessels, feeding the tumor. "We sometimes find true pus in these sacks, at a certain stage; consequently, according to the opinions of some modern pathologists, it should be furnished from the circulation."—*Cohnheim.*

M. Vulpian, Huyem, and others, regard pus globules as the colorless blood corpuscles, though still in doubt with some, they may with equal propriety be regarded as decolorised blood disks, the result of local action of the inflamed tissue on the blood stasis, or during its slow movement through the diseased part. When the pulp in a tooth dies from arsenic, there is a dead point in contact with a living tissue. When the tendency to diseased action is great the blood vessels are cut off, as well as the nerves, and there must be set up a line of demarcation, and a new or healing and reparatory process, which may be of a healthy or unhealthy nature, and abscess or no abscess is the question at issue. It is not always the case that true abscess characterizes the diseased condition of the alveolus after devitalization of teeth; on the contrary, it forms the exception, not the rule. Morbid growth of cellular tissue more frequently follow dead teeth. In cases like the above, it would be exceedingly difficult to diagnose, and more so to treat.

The tooth spoken of above I intended saving as a dental souvenir, but it was carried away by mice under the floor, where I have no doubt they held high carnival, not knowing the scientific value of their repast.

